# Integrated miRNA-mRNA Atlas Reveals Temperature-Graded Brain Neuroendocrine Adaptation to Cold Stress in Silvery Pomfret (*Pampus argenteus*)

**DOI:** 10.3390/biology14091265

**Published:** 2025-09-13

**Authors:** Danqing Yin, Xianhui Ning

**Affiliations:** 1 College of Marine Science and Engineering, Jiangsu Province Engineering Research Center for Aquatic Animals Breeding and Green Efficient Aquacultural Technology, Jiangsu Key Laboratory of Ocean-Land Environmental Change and Ecological Construction, Nanjing Normal University, Nanjing 210023, China; 2 School of Biomedical Sciences, Li Ka Shing Faculty of Medicine, The University of Hong Kong, Pokfulam, Hong Kong SAR 999077, China; u3009041@connect.hku.hk

**Keywords:** cold stress, microRNA, *Pampus argenteus*, adaptation strategy, miRNA-mRNA network

## Abstract

Temperature stress is a major cause of mortality in aquaculture, yet the molecular mechanisms underlying cold adaptation in commercially important fish such as the silver pomfret (*Pampus argenteus*) remain poorly understood. In this study, we used integrated miRNA and mRNA transcriptomics to analyze brain tissue responses of silver pomfret under gradient cold exposure conditions (28 °C control, 18 °C moderate, 13 °C extreme). We identified 85 differentially expressed miRNAs (DEmiRs), with 22 altered under moderate cold and 68 altered in extreme cold, demonstrating that miRNA regulatory activity intensifies with decreasing temperature. Combined miRNA target prediction and expression correlation analysis revealed 8 and 247 differentially expressed target genes (DETGs), which formed cold-adaptive regulatory networks with corresponding DEmiRs. Functional analysis showed enrichment of pathways related to circadian rhythm (e.g., PER targeted by miR-429-y and miR-181-z), immunity (e.g., JUN-miR-10545-x cluster), and endocrine function (e.g., NHERF1-miR-181-z). Notably, miR-181-z was identified as a central regulatory hub, interacting with 13.2% nodes in BE network. Our study provides the first comprehensive miRNA-mRNA network resource for cold stress response in silver pomfret, offering valuable molecular biomarkers for breeding cold-resilient strains and enhancing sustainable aquaculture practices.

## Simple Summary

Silver pomfret (*Pampus argenteus*), an economically vital aquaculture species, suffers high mortality during winter cold events, yet the molecular basis of its cold adaptation remains unclear. To address this, we exposed fish to control (28 °C), moderate cold (18 °C), and extreme cold (13 °C) conditions and analyzed brain regulatory networks involving miRNAs, small molecules that fine-tune gene expression. We discovered that extreme cold induced 68 key miRNAs (triple the response at 18 °C), with miR-181-z acting as a master switch controlling circadian rhythms (via PER1), hormone balance (NHERF1, Notch1a), and immune defenses (BHLHE40). While moderate cold caused minimal disruption, extreme cold severely impaired endocrine and immune functions. Crucially, we identified 18 °C as a safe temperature threshold to prevent hormonal damage in aquaculture and propose miR-181-z as a promising biomarker for breeding cold-resistant strains. These insights help reduce winter losses in fisheries and enhance climate resilience for sustainable seafood production.

## 1. Introduction

Temperature constitutes a fundamental abiotic variable that governs physiological homeostasis in ectothermic vertebrates, particularly teleosts whose metabolic processes exhibit pronounced thermal dependence [1]. Aquatic environments subject fish to dynamic thermal regimes through diurnal oscillations and seasonal transitions [2,3], yet rapid cooling events can precipitate catastrophic mortality in both natural populations and aquaculture systems. This vulnerability underscores the imperative to decipher molecular adaptation mechanisms to cold stress. Cellular responses to thermal challenge include compromised mitochondrial oxidative phosphorylation, altered membrane lipid composition affecting fluidity, cytoskeletal disorganization, and elevated production of molecular chaperones such as HSP70/90 [4,5,6]. These perturbations cascade into organ dysfunction, impairing brain function, hepatic detoxification, branchial gas exchange, neuromuscular coordination, and muscular contractility [7], collectively threatening organismal viability under thermal extremes.

MicroRNAs (miRNAs) represent a class of ~22-nucleotide non-coding RNAs that orchestrate post-transcriptional gene silencing through mRNA destabilization and translational repression, thereby fine-tuning diverse physiological pathways [8]. Emerging evidence implicates miRNAs as master regulators of cold adaptation, with their diagnostic and therapeutic potential increasingly recognized across taxa [9,10]. Mammalian studies reveal intricate miRNA-mediated cold responses. Brown adipose tissue-derived miR-378a-3p enhances hepatic gluconeogenesis during non-shivering thermogenesis by suppressing phosphatidylinositol 3-kinase catalytic subunit p110α, a mechanism validated by impaired cold tolerance in miR-378^-/-^ mice and rescued through targeted miR-378a-3p restoration [11]. Conversely, cardiopulmonary pathologies like cold-aggravated pulmonary hypertension involve dysregulation of inflammation-associated miRNAs (e.g., miR-146a-5p and miR-155-5p) that amplify cytokine cascades [12]. While piscine miRNA research under cold stress remains less comprehensive, zebrafish models demonstrate conserved functions: miRNAs modulate genomic plasticity during cold shock [13], and mediate ovarian dysfunction through endocrine disruption [14]. Collectively, these evolutionarily conserved miRNA-mRNA networks constitute critical components of vertebrate cold stress adaptation.

The silvery pomfret (*Pampus argenteus*), a widely distributed marine fish in China, has become a focus of aquaculture research due to declining wild stocks from overfishing [15]. This depletion has accelerated aquaculture development efforts, with considerable advances achieved in captive breeding and husbandry protocols. Current research priorities predominantly address production bottlenecks, including bacterial pathogenesis [15,16,17,18], genetic structure [19,20], and osmotic regulation [21]. Nevertheless, a critical knowledge gap persists regarding molecular adaptation mechanisms, particularly miRNA-mediated responses to cold stress, despite this species’ documented vulnerability to seasonal temperature fluctuations in its native habitat.

In this study, we implemented integrated whole-transcriptome sequencing to delineate temperature-responsive miRNA-mRNA regulatory circuitry in the brain of *Pampus argenteus* exposed to gradient cold challenges (control: 28 °C; moderate cold: 18 °C; extreme cold: 13 °C). We identified differentially expressed miRNAs (DEmiRs) responsive to cold stress and characterized their dynamic changes. Target genes of these DEmiRs (DETGs) were predicted based on miRNA-mRNA interaction analyses and expression correlations. Functional enrichment analyses of DETGs were performed, and cold stress-associated DEmiR-DETG regulatory networks were constructed and analyzed. This work provides the first comprehensive profile of miRNAs in cold-stressed silver pomfret, enhancing our understanding of the complex molecular networks underlying cold adaptation in fish.

## 2. Materials and Methods

### 2.1. Ethics Statement

All of the experimental procedures in the present study were performed according to the guidelines for the Care and Use of Laboratory Animals in China, and were approved by the Nanjing Normal University Animal Ethics Committee (permit No. SYXK2015-0028).

### 2.2. Experimental Design and Sample Collection

Clinically healthy, wild-type juvenile silvery pomfret (approximately 5 months old, 7.3 ± 1.2 g in body weight) were obtained from a large population at the Jiangsu Marine Fisheries Research Institute (Rudong base, Nantong, China) in July 2024. A total of 90 fish were acclimatized for one-week at 25 °C in recirculating aquaculture systems. Subsequently, they were randomly distributed into three experimental groups (n = 30 per group), each held in three replicate tanks (10 fish per tank). Control (BC) group was maintained at 25 °C, moderate cold stress (BM) group was acclimated to 18 °C, and extreme cold stress (BE) group was acclimated to 13 °C. To mitigate the potential for thermal shock induced by rapid cooling, the water temperature was gradually lowered at a rate of 0.85 °C/h, consistent with methodologies established in previous study [22]. After 96 h of temperature exposure, fish were euthanized by immersion in MS-222 (Sigma, St. Louis, MO, USA) [23], followed by cerebral dissection under sterile conditions. Brain tissues were flash-frozen in liquid nitrogen and stored at −80 °C pending RNA extraction.

### 2.3. mRNA and miRNA Sequencing and Analysis

#### 2.3.1. Library Construction and Sequencing

Transcriptome profiling was performed following established methodologies [24,25]. Briefly, total RNA was isolated from brain tissues of control and experimental groups using TRIzol reagent (Invitrogen, Carlsbad, CA, USA). RNA integrity was verified (RIN > 8.0) prior to library construction. Strand-specific mRNA libraries and small RNA libraries were prepared according to Illumina TruSeq protocols. Sequencing was conducted on separate platforms: mRNA libraries on Illumina HiSeq™ 4000 (Illumina, San Diego, CA, USA), and miRNA libraries on Illumina HiSeq XTen. Quality-controlled processing removed adapter sequences, low-quality bases (Q-score < 20), and reads shorter than 18 nt. High-quality reads were mapped to *Pampus argenteus* genome assembly (PRJNA1066130) using Bowtie v1.1.2 [26], or aligned against the GeneBank and Rfam databases via Blastall (v2.2.25) as previous reported [27]. The mRNAs were identified using TopHat2 (v2.1.1), followed by transcript assembly and quantification via Cufflinks (v2.2.1) [28] with fragment bias correction. Known miRNAs were annotated against miRBase v21.0, with novel miRNAs predicted through MIREAP (v0.2) [29] using default hairpin structure parameters.

#### 2.3.2. Differential Expression Analysis

Transcript quantification was performed using standardized normalization methods [24]: mRNA expression levels were calculated as Fragments Per Kilobase per Million mapped reads (FPKM), while miRNA abundance was determined by tags per million (TPM). Differential expression analysis was subsequently conducted employing the edgeR package (v3.12.1) [30], with statistical significance thresholds established at Log2|FC| > 1 and *p* < 0.05. These criteria identified differentially expressed genes (DEGs) and miRNAs (DEmiRs) across experimental groups.

#### 2.3.3. Target Genes Identification and Functional Enrichment

Target prediction for differentially expressed miRNAs (DEmiRs) employed a consensus approach using three complementary algorithms: RNAhybrid (v2.1.2) paired with svm_light (v6.01), Miranda (v3.3a), and TargetScan (v7.0) [27]. Genes predicted by all three tools were designated candidate targets. Subsequent expression correlation analysis integrated miRNA-seq and mRNA-seq data, retaining only candidates exhibiting both differential expression and significant negative correlation with corresponding DEmiRs as high-confidence differentially expressed target genes (DETGs). Functional enrichment of DETGs was performed against Gene Ontology (GO) and Kyoto Encyclopedia of Genes and Genomes (KEGG) databases using hypergeometric testing to identify significantly overrepresented biological terms and pathways.

#### 2.3.4. Regulatory miRNA-mRNA Network Construction

miRNA-mRNA interactions were integrated to construct cold stress-responsive regulatory networks using Cytoscape (v3.7.1) [31]. Networks were visualized with differentially expressed miRNAs (DEmiRs) as source nodes and their corresponding target genes (DETGs) as target nodes, with edge weights representing significant negative expression correlations (*r* < −0.7, *p* < 0.05).

### 2.4. Experimental Validation

To validate sequencing results, we performed qRT-PCR analysis on six randomly selected miRNAs and six mRNAs exhibiting significant differential expression. Reactions utilized SYBR Green Master Mix (Hieff UNICON, Shanghai, China) on an ABI StepOne Plus system (Applied Biosystems, Foster, CA, USA) following manufacturer protocols. Expression quantification employed the 2^−ΔΔCt^ comparative Ct method [32], with primer sequences detailed in Table A1. Correlation between sequencing and qRT-PCR results was statistically evaluated using Pearson’s method in R (v3.5.2).

## 3. Results

### 3.1. miRNA Sequencing and Identification

**Figure 1:**
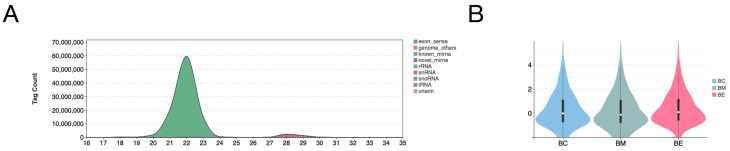
Characterization of brain miRNAs in cold-stressed silver pomfret. (**A**) Length distribution profile of identified miRNAs. (**B**) Expression patterns of miRNAs across experimental groups. visualized by violin plot. Group designations: BC (control, 28 °C), BM (moderate cold stress, 18 °C), BE (extreme cold stress, 13 °C).

To investigate the effects of cold stress on miRNA expression in silver pomfret, we performed high-throughput miRNA sequencing. A total of 108,229,469 clean reads were generated. Following quality control, 99.38% of reads were identified as high-quality clean reads and retained for analysis (Table 1). After removing the reads lacking insert sequence or with ploy A, 103,722,881 clean tags were used for miRNAs detection. This analysis identified 506 known miRNAs and discovered 185 novel miRNAs in silvery pomfre, representing the first report of these novel sequences. The combined 691 miRNAs exhibited a characteristic length distribution predominantly at 22 nt (20–23 nt range), with a minor secondary peak observed at approximately 28 n (Figure 1A). A heatmap revealed distinct expression patterns for these miRNAs across experimental groups (Figure 1B).

### 3.2. Cold Stress Induces Significant Brain miRNA Responses

**Figure 2:**
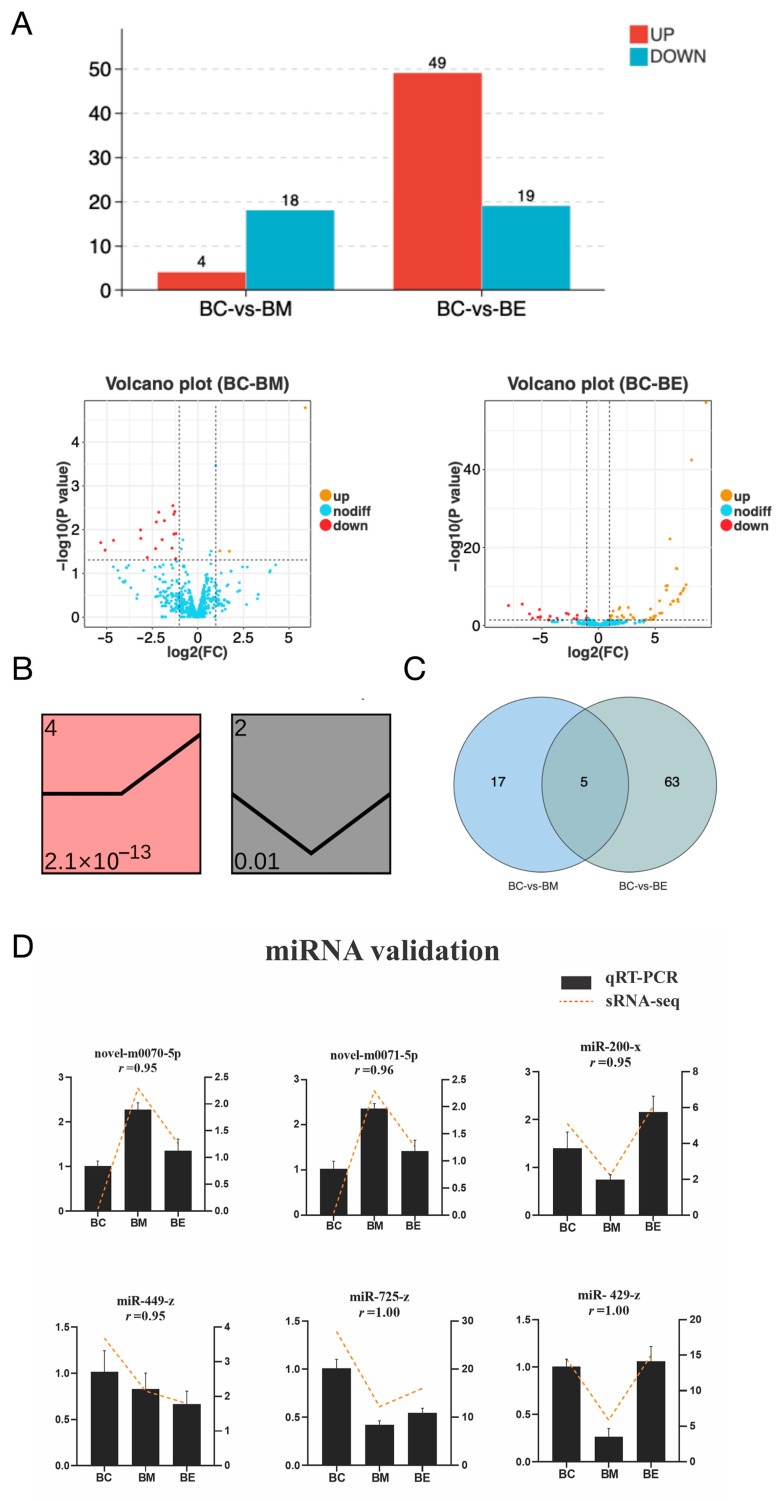
Dynamics of cold-responsive miRNAs in silver pomfret brain. (**A**) Differentially expressed miRNAs (DEmiRs) in moderate cold (BM, 18 °C) and extreme cold (BE, 13 °C) versus control (BC, 28 °C). (**B**) Temporal expression profiles of DEmiRs clustered by trend analysis. Squares represent significantly enriched expression patterns (*p* < 0.01), with profile IDs (top) and statistical significance (bottom) indicated. (**C**) Venn diagram of DEmiR overlap between experimental groups. (**D**) Validation of sRNA-seq results by qRT-PCR for six representative DEmiRs. Results are shown as means ± standard deviation (n = 3).

Following cold exposure, a total of 85 differentially expressed miRNAs (DEmiRs) were identified in silver pomfret brain tissue. Specifically, the moderate cold stress group (BM) exhibited 22 DEmiRs (4 upregulated and 18 downregulated; Figure 2A), while the extreme cold stress group (BE) showed a greater response with 68 DEmiRs (49 upregulated and 19 downregulated; Figure 2A). This progressive increase in DEmiR numbers with decreasing temperature indicates an intensification of the miRNA regulatory response under more severe cold stress. To characterize the dynamic expression patterns of these DEmiRs across stress levels, trend analysis was performed. The DEmiRs were significantly clustered (*p* < 0.01) into two distinct expression profiles (Figure 2B). Profile 4 displayed stable expression from the control group (BC) to BM, followed by increased expression from BM to BE (Figure 2C). Conversely, Profile 2 exhibited decreased expression from BC to BM and increased expression from BM to BE (Figure 2C). Analysis of DEmiR distribution revealed that five miRNAs (miR-300-y, miR-434-y, miR-674-y, novel-m0070-5p, and novel-m0071-5p) were differentially expressed in both BM and BE groups (Figure 2C). Seventeen DEmiRs, including representative examples such as miR-429-y, miR-725-z, and miR-200-x, were exclusively altered in the BM group (Figure 2C). Sixty-three DEmiRs were specific to the BE group, including miR-181-z, novel-m0128-5p, miR-674-y, miR-150-x, miR-300-y, miR-449-z, miR-10545-x, miR-276-y, miR-252-x, miR-2284-z, miR-14-y, miR-1843-x, miR-340-x, miR-1298-x, miR-1198-x, miR-3477-x, and miR-3785-y (Figure 2C). The differential expression of six representative DEmiRs (novel-m0070-5p, novel-m0071-5p, miR-200-x, miR-449-Z, miR-725-z and miR-429-z) was further validated using qRT-PCR. The qRT-PCR results showed strong concordance with the sRNA-seq data, with correlation coefficients reaching 1.00 (Figure 2D).

### 3.3. Integrated Analysis of miRNA-mRNA During Cold Stress

**Figure 3:**
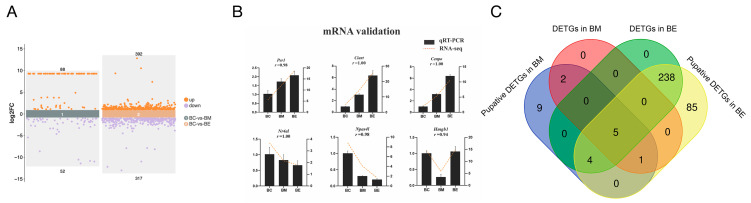
Identification of the differentially expressed target genes of DEmiRs (DETGs) in cold-stressed silver pomfret. (**A**) Volcano plots of differentially expression genes (DEGs) in brain tissues under moderate cold (BM, 18 °C) and extreme cold (BE, 13 °C) versus control (BC, 28 °C). (**B**) Validation of RNA-seq results by qRT-PCR for six representative DEGs. Results are shown as means ± standard deviation (n = 3). (**C**) Venn diagram showing the DETGs identification processes.

To identify potential target genes of the DEmiRs, we first performed transcriptome sequencing on silver pomfret brain tissue under cold stress conditions. This analysis identified 140 differentially expressed genes (DEGs) in BM and 709 DEGs in BE (Figure 3A). The expression patterns of six randomly selected DEGs were validated by qRT-PCR, showing high correlation coefficients (ranging from 0.94 to 1.00) with the RNA-seq results, confirming the reliability of the transcriptome data (Figure 3B). We then conducted an integrated miRNA-mRNA co-analysis to identify regulatory relationships. Putative targets of the DEmiRs were predicted using overlapping results from three computational methods. This initial prediction yielded 21 candidate target genes for BM-associated DEmiRs and 333 for BE-associated DEmiRs. To increase stringency, we performed expression correlation analysis between these candidate target genes and their corresponding DEmiRs. Genes exhibiting both differential expression and a significant negative correlation with their targeting DEmiRs were considered high-confidence targets (DETGs). This stringent filtering identified 8 DETGs for the BM group and 247 DETGs for the BE group. Notably, five DETGs, including PER1, CIART, CENPO, NR4A1, and NPAS4L, were common to both cold stress groups (Figure 3C).

### 3.4. Functional Landscapes of DEmiR-Mediated Pathways

**Figure 4:**
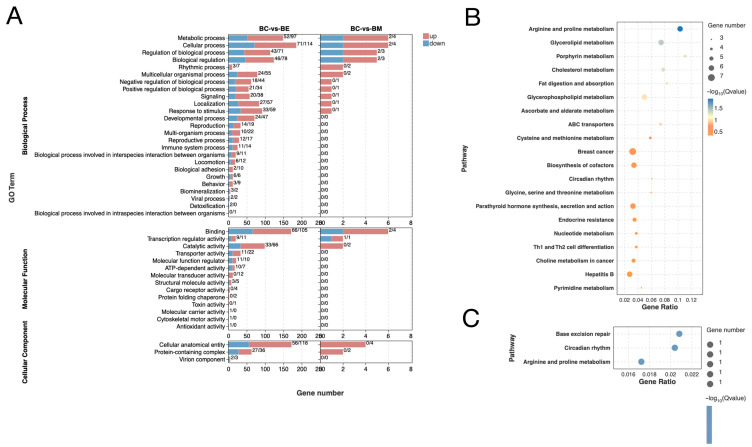
Gene Ontology (GO) (**A**) and Kyoto Encyclopedia of Genes and Genomes (KEGG) (**B**,**C**) functional enrichment of differentially expressed target genes of DEmiRs (DETGs).

To elucidate the biological functions influenced by cold stress-induced DEmiRs, we performed GO and KEGG functional enrichment analyses on the DETGs. GO analysis revealed both shared and stress-intensity-specific enriched terms. Biological processes including metabolic process, rhythmic process and response to stimulus, along with molecular functions, such as binding, transcription regulator activity and catalytic activity, were significantly enriched in both BM and BE groups, though enrichment was markedly stronger in the BE group (Figure 4A). Conversely, BE-specific processes included developmental, reproductive, immune system, growth and behavior. Similarly, molecular functions enriched exclusively in BE included transporter activity, ATP-dependent activity and antioxidant activity (Figure 4A). Cellular component analysis showed enrichment for cellular anatomical entity and protein-containing complex in both groups, while virion component was uniquely enriched in BE (Figure 4A).

KEGG pathway analysis identified only three significantly enriched pathways in BM, including base excision repair, circadian rhythm and arginine and proline metabolism (Figure 4B). In contrast, BE induced significant enrichment in 63 pathways. The top 20 pathways (Figure 4C) included lipid metabolism (glycerolipid, cholesterol, fat digestion and absorption and glycerophospholipid), immune regulation (Th1 and Th2 cell differentiation, hepatitis B and breast cancer), and endocrine function (parathyroid hormone synthesis, secretion and action, and endocrine resistance). Collectively, these enrichment results demonstrate that cold stress perturbs diverse biological processes, with extreme low temperature exhibiting particularly pronounced effects on immune and endocrine pathways.

### 3.5. Cold-Responsive miRNA-mRNA Regulatory Networks

**Figure 5:**
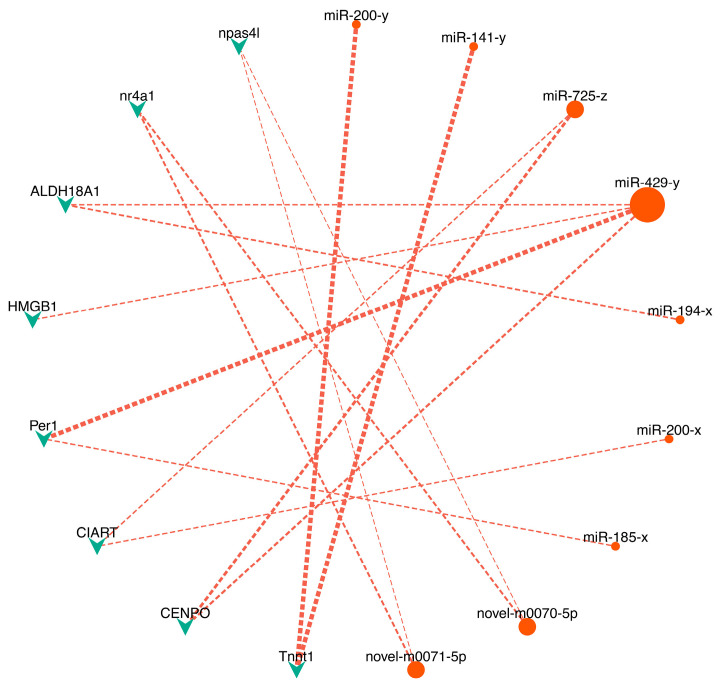
Regulatory network of miRNA-mRNA interactions in brain under moderate cold stress (BM, 18 °C). The red round nodes and green triangle nodes indicate differentially expressed miRNAs (DEmiRs) and target genes (DETGs) induced by cold stress, respectively.

**Figure 6:**
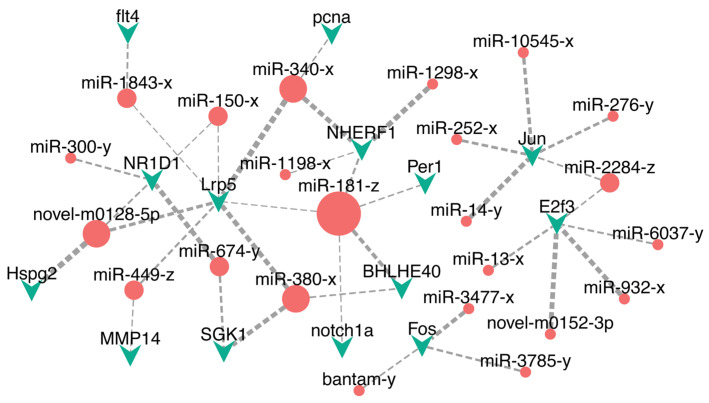
Functional regulatory network underlying extreme cold adaptation (BE, 13 °C) in silver pomfret brain. Integrated miRNA-mRNA interactions associated with circadian rhythm, immunity, and endocrine pathways. The red round nodes and green triangle nodes indicate differentially expressed miRNAs (DEmiRs) and target genes (DETGs) induced by cold stress, respectively.

To elucidate the regulatory interactions between miRNAs and mRNAs during cold stress, we constructed DEmiR-DETG networks for each stress group. The BM network comprised 9 DEmiRs and 8 DETGs forming 15 regulatory pairs (Figure 5), while the BE network involved 65 DEmiRs and 247 DETGs connected by 600 pairs. Focusing on pathways implicated by functional enrichment, we visualized key regulatory pairs associated with circadian rhythm, immunity, and endocrine functions, which collectively involved 23 DEmiRs and 14 DETGs, forming 38 regulatory pairs (Figure 6). In the BM network, miR-429-y emerged as the predominant hub miRNA, exhibiting the highest connectivity through interactions with DETGs including PER1 and HMGB1 (Figure 5). Additional significant interactions in this network included CIART regulation by miR-725-z and miR-200-x. Within the more complex BE network, miR-181-z functioned as the primary hub miRNA, demonstrating extensive connectivity with targets such PER1, NHERF1, and BHLHE40 (Figure 6). Key regulatory relationships in this network also included NR1D1 regulated by novel-m0128-5p, miR-674-y, miR-150-x and miR-300-y; MMP14 targeting by miR-449-z; Jun regulation through miR-10545-x, miR-276-y, miR-252-x, miR-2284-z and miR-14-y; FLT4 targeting by miR-1843-x; NHERF1 regulation by miR-181-z, miR-340-x, miR-1298-x and miR-1198-x; Fos regulation by bantam-y, miR-3477-x and miR-3785-y (Figure 6).

## 4. Discussion

miRNAs are increasingly recognized as critical molecular regulators of cold stress adaptation in fish [33]. Our integrated micro-transcriptome and transcriptome analysis of silvery pomfret exposed to graded cold stress revealed 85 DEmiRs, demonstrating the complexity of miRNA-mediated responses to thermal challenge. Notably, 80% of these DEmiRs were specific to extreme cold stress (BE, 13 °C), reflecting intensified miRNA regulation at lower temperatures. Conversely, the moderate cold group (BM, 18 °C) still elicited significant responses, with >20% of DEmiRs uniquely altered after 96 h exposure, which was consistent with acute cold stress patterns observed in zebrafish [13]. The threefold greater DEmiR count in BE versus BM underscores a temperature-dependent escalation of molecular reprogramming, further evidenced by broader functional enrichment of DETGs in BE. Within shared biological categories, DETG numbers were consistently higher under extreme cold, collectively affirming miRNAs as pivotal orchestrators of piscine cold stress responses. Taken together, these results highlight the importance of miRNAs in fish responses to cold stresses.

Cold stress adaptation involves intricate miRNA-gene regulatory networks. Our study deciphered this interplay through constructed DEmiR-DETG networks for both stress intensities. The BM network (9 DEmiRs-8 DETGs) and expanded BE network (65 DEmiRs-247 DETGs) collectively modulate core physiological processes, particularly circadian rhythm, immunity, and endocrine functions, highlighting system-wide molecular adjustments to thermal challenge.

### 4.1. Circadian Rhythm

Circadian rhythms represent evolutionarily conserved physiological and behavioral adaptations to 24 h environmental cycles, governed by complex interplay between external zeitgebers (e.g., light-dark cycles, thermal fluctuations) and endogenous molecular oscillators comprising transcription-translation feedback loops [34]. Our study provides compelling evidence that cold stress fundamentally reprograms circadian regulatory networks in silvery pomfret, with circadian entrainment pathways significantly enriched among differentially expressed miRNA targets across both moderate (BM, 18 °C) and extreme cold (BE, 13 °C) exposure groups. This perturbation mirrors mammalian models where chronic cold exposure induces phase-shifting of hepatic metabolic gene expression and alters circadian transcriptome dynamics [35], suggesting deep evolutionary conservation of temperature-sensitive clock mechanisms. Central to this thermal entrainment is Per which we identified as a key nodal point targeted by distinct miRNAs under graded cold stress: miR-429-y in BM and miR-181-z in BE. Mammalian homologs PER1 and PER2 constitute core components of the suprachiasmatic nucleus (SCN) pacemaker, where they regulate thermoregulatory circuits through hypothalamic-autonomic nervous system connections [36,37]. The cold hypersensitivity observed in *Per2*^-/-^ mice, where it attributed to impaired brown adipose tissue thermogenesis and uncoupling protein 1 (UCP1) dysregulation [37], underscoring the essential role of PER proteins in cold adaptation. The significant *PER1* upregulation we detected in silver pomfret brain tissue implies compensatory conservation of this protective mechanism in teleosts, likely facilitated through temperature-dependent suppression of its targeting miRNAs. This graduated regulatory strategy (miR-429-y attenuation at 18 °C vs. miR-181-z inhibition at 13 °C) may represent an evolutionary innovation enabling fine-tuned thermal responses across ecologically relevant temperature ranges. Beyond PER1, we identified two additional regulatory axes: *CIART*, a modulator of BMAL1/CLOCK-driven transcription [38] targeted by miR-725-z and miR-200-x exclusively under moderate cold stress, and NR1D1, a heme-responsive repressor of circadian output targeted in extreme cold by a miRNA consortium including novel-m0128-5p, miR-674-y, miR-150-x and miR-300-y. The latter finding is particularly significant as cold-induced NR1D1 remodeling in murine peripheral tissues coordinates metabolic reprogramming through PPARγ signaling [39], suggesting our observed miRNA-NR1D1 interactions may gatekeep similar adaptive responses in fish. Collectively, these regulatory circuits demonstrate how cold stress co-opts hierarchical miRNA-circadian networks across vertebrates, with silver pomfret exhibiting both conserved elements (*PER1* and *NR1D1*) and taxon-specific innovations (temperature-graded miRNA switching) to maintain chronobiological homeostasis under cold challenge.

### 4.2. Immune Response

Cold stress is a well-established immunosuppressive factor in poikilothermic vertebrates, with particular significance for aquatic species like fish that experience direct thermal exchange with their environment [22,40]. The inherent vulnerability of teleosts is compounded by their constant exposure to aquatic pathogens, where depressed immune function at low temperatures creates a critical survival challenge. Our findings substantiate this paradigm, revealing pronounced cold-induced immunomodulation in silvery pomfret, especially under extreme cold exposure (BE, 13 °C). Pathway enrichment analysis demonstrated significant perturbation of immune-related pathways including hepatitis B and breast cancer signaling, mammalian disease models that serve as valuable proxies for understanding fundamental immune dysregulation mechanisms in fish. Notably, the increased expression of miR-449-z, a regulator of matrix metalloproteinase MMP14, suggests impaired extracellular matrix homeostasis. Given MMP14’s dual role in breast cancer metastasis and neuroinflammation through blood–brain barrier modulation [41], its dysregulation may indicate compromised neuroimmune competence in fish brain tissue during severe cold stress.

Further evidence of immune disruption emerged through inflammasome pathway components. We identified five miRNAs (miR-10545-x, miR-276-y, miR-252-x, miR-2284-z, miR-14-y) collectively targeting JUN, a transcriptional activator of NLRP3 inflammasome assembly. This complex, when activated during viral challenges in mammals, initiates pyroptotic cell death and propagates inflammatory cascades [42]. The parallel involvement of FLT4 (targeted by miR-1843-x) extends this mechanism to bacterial defense contexts, as demonstrated in murine models where FLT4-mediated inflammasome activation coordinates antibacterial immunity [43]. Significantly, inflammatory dysregulation was not exclusive to extreme cold. n moderate cold conditions (BM, 18 °C), miR-429-y mediated regulation of HMGB1, a damage-associated molecular pattern (DAMP) molecule released during necroinflammation that amplifies innate immune responses through TLR4/NF-κB signaling [44]. The hierarchical suppression of these immunoregulatory miRNAs across cold stress intensities suggests a temperature-dependent breakdown of inflammatory containment mechanisms. Collectively, these multi-layered disruptions, from pathogen recognition to cytokine amplification, delineate a compromised immunophenotype in cold-stressed silver pomfret, potentially explaining their heightened disease susceptibility in winter aquaculture conditions.

### 4.3. Endocrine Adaptation Strategy

The endocrine system serves as a master regulator of growth, reproduction, and metabolic homeostasis across vertebrates, making its integrity critical for environmental adaptation [45,46]. Our study reveals temperature-dependent endocrine disruption in silvery pomfret, where extreme cold exposure (BE, 13 °C) triggered significant dysregulation absent in moderate conditions (BM, 18 °C). This finding aligns with zebrafish studies demonstrating complete spawning inhibition below 18 °C through hypothalamic suppression of gonadotropin-releasing hormone (GnRH) and insulin signaling pathways [47], while mammalian models show chronic cold stress activates the hypothalamus–pituitary–adrenal axis to elevate corticosteroid levels [48]. The absence of endocrine perturbations at 18 °C in our model suggests silver pomfret may possess compensatory mechanisms within this thermal range.

Molecular analysis identified two pivotal regulators of endocrine function in extreme cold. NHERF1 was targeted by miR-181-z, miR-340-x, miR-1298-x and miR-1198-x. As an established biomarker of endocrine resistance in luminal B breast cancer [49], NHERF1 coordinates hormone receptor trafficking and G-protein signaling. Its dysregulation implies compromised steroid sensitivity. Fos was regulated by bantam-y, miR-3477-x and miR-3785-y. This transcription factor drives endocrine therapy resistance in mammary carcinomas through AP-1 [50]. Enrichment of breast cancer and endocrine resistance pathways among BE-associated DEmiRs further substantiates conserved endocrine disruption mechanisms between mammalian disease states and piscine cold stress. Notably, we identified Notcha1a targeting by miR-181-z, a pathway essential for pancreatic endocrine cell differentiation in zebrafish, where Notch suppression causes precocious NeuroD-positive cell maturation in pancreatic ducts [51]. This regulatory axis may represent an adaptive mechanism for metabolic reprogramming during cold stress. The centrality of miR-181-z warrants special emphasis: as the top network hub in BE, it concurrently regulates NHERF1, Notch1a, and circadian gene PER1. This multifunctionality mirrors its role in rainbow trout antiviral defense against infectious hematopoietic necrosis virus [52], suggesting miR-181-z serves as an evolutionary conserved stress integrator coordinating endocrine, immune, and circadian adaptation. The convergence of these pathways through a single miRNA highlights sophisticated regulatory efficiency in cold stress response, potentially offering biomarkers for aquaculture management where endocrine dysfunction compromises reproductive success and growth performance in winter conditions.

### 4.4. Potential Applications

RNA-based therapeutics, which utilize RNA molecules to modulate gene expression and protein function, represent a rapidly advancing frontier in biomedicine due to their high specificity, minimal genotoxicity, and capacity for sustained efficacy, particularly in the treatment of genetically defined and rare diseases [53]. NcRNAs, including miRNAs, have attracted significant interest as promising therapeutic agents [54,55,56]. For example, ginger-derived exosome-like nanoparticles (GELNs) deliver osa-miR164d, which reprograms macrophage polarization and inhibits intestinal inflammatory responses [57]. In another study, miR-148a-3p was selected as a therapeutic target for uterine leiomyomas, which showed that both local and systemic delivery of an anti-miR-148a-3p LNA (locked nucleic acid-based inhibitors) significantly reduced tumor volume and cell proliferation [58].

These advances highlight the broader potential of RNA therapeutics, which may be extendable beyond human medicine into agricultural and aquaculture contexts. In this study, we identified several key molecular regulators of cold stress response, most notably the hub miRNA miR-181-z and its downstream targets (e.g., Per1, NHERF1). These molecules represent valuable candidates for applied strategies such as: (1) Molecular marker-assisted breeding: miR-181-z and related biomarkers could be incorporated into screening programmes to identify and select individuals with enhanced innate cold tolerance, accelerating the development of resilient silver pomfret strains. (2) Dietary RNA interventions: Synthetic mimics of miR-181-z, or inhibitors of its stress-related target genes, could be developed as RNA-based feed additives to enhance cold acclimation and reduce winter mortality under production conditions. (3) Temperature management guidelines: Our finding that 18 °C serves as a critical threshold for endocrine and immune disruption offers a science-based target for aquaculture management. Maintaining water temperatures above this threshold during cold events may prevent systemic dysfunction and significantly improve survival rates. Together, these approaches illustrate how molecular insights into cold stress response can be translated into practical interventions, ranging from genetic improvement to environmental management, that enhance productivity, welfare, and sustainability in silver pomfret aquaculture.

## 5. Conclusions

This integrated transcriptomic analysis elucidates the fundamental role of miRNA-mRNA regulatory networks in mediating brain neuroendocrine adaptation to cold stress in *Pampus argenteus*, revealing a hierarchical response architecture calibrated to thermal severity. We demonstrate that extreme cold exposure (13 °C) triggers extensive miRNA reprogramming, mobilizing 68 differentially expressed miRNAs, triple the response observed at moderate stress (18 °C), with miR-181-z emerging as a central orchestrator of circadian (*PER1*), endocrine (*NHERF1*, *Notch1a*), and immune (BHLHE40) pathways. Crucially, evolutionarily conserved mechanisms governing inflammasome activation, endocrine resistance, and circadian entrainment underpin cold adaptation, evidenced by striking functional parallels between piscine and mammalian stress responses. The temperature-dependent suppression of regulatory miRNAs enables precise tuning of neuroendocrine outputs, while novel species-specific regulators like novel-m0128-5p highlight adaptive innovations. These findings also provide miR-181-z as a potential biomarker for breeding thermal-resilient strains. Collectively, these findings establish miRNAs as pivotal regulators of piscine cold adaptation, providing a mechanistic framework for future exploration of miRNA-mediated stress responses in teleosts.

## Figures and Tables

**Table 1:** Summary of data processing of micro-transcriptome libraries in silvery pomfret in response to cold stress.

Sample	Clean Reads	HQ Reads	HQ Ratio (%)	Clean Tags	Known miRNA Abundance	Novel miRNA Abundance
BC-1	11577604	11412232	98.57	11047812	10455376	7072
BC-2	12040977	11974548	99.45	11437905	10604543	8307
BC-3	12925738	12863379	99.52	12556139	12228779	9526
BM-1	10779702	10731401	99.55	10414358	9969297	7680
BM-2	12155435	12100330	99.55	11594623	10857439	8139
BM-3	11946167	11872926	99.39	11498661	10947635	10184
BE-1	12498393	12429937	99.45	11984423	10763958	10113
BE-2	12408964	12340518	99.45	11693420	10590040	9840
BE-3	11896489	11831975	99.46	11495540	11078132	10806

HQ reads, high quality reads. “BC”, “BM”, and “BE” indicate the fish Brain in Control (25 °C) group, Medium (18 °C) and Extreme (13 °C) cold stress group, respectively.

## Data Availability

The original contributions presented in this study are included in the article. Further inquiries can be directed to the corresponding author.

## Appendix A

**Table A1:** Summary of primers used for qRT-PCR validation.

Name	Primer (5′ to 3′)	Primer Tm	Product Length	Accession Number	Amplification Efficiency
novel-m0070-5p	CCCTGTGGTACCTGTACTACCTG	56.7	/	/	/
novel-m0071-5p	GCCTGTGGTACCTGTACTACCT	56.7	/	/	/
miR-429-y	GCCGTAATACTGTCTGGTAATGCCG	51.4	/	/	/
miR-725-z	CGCTTCAGTCATTGTTTCTGGTCGT	56.6	/	/	/
miR-200-x	GCATCTTACCTGACAGTGCTGGA	60.6	/	/	/
miR-449-z	ATTATTGGCAGTGTACTGCTGCTGG	60.1	/	/	/
Nr4a1	CATCGGATGCACGATTAG	50.4	201	evm.TU.Chr04.473	90.01%
	TGTGGGAGATTGGTCAGG	54.2			
Npas4	TTCACCGCATGAACCTAG	51.8	197	evm.TU.Chr12.134	106.95%
	TACCCAGAAGACGAGAAAGATA	51.8			
Hmgb1	TGATTACCCTGGGCTTTC	51.6	172	evm.TU.Chr15.666	93.12%
	TGATGCCGTATCCACTTTA	50.1			
Per1	CGGAAGGTGGCGTTTATT	52.7	155	evm.TU.Chr01.395	105.80%
	ACGGGCTGTACCAGGAGA	58.7			
Ciart	CTTTGCTTGGGCTTCTAC	65.8	232	evm.TU.Chr20.664	106.27%
	GGAGTGATGGTCTGGGTT	54.2			
Cenpo	TGACCTGAAGCCAACTCT	53.0	232	evm.TU.Chr14.568	106.02%
	CAGCACCAATACTGAACATA	49.3			
U6	TGACCTGAAGCCAACTCT	59.4	/		107.99%
	AACGCTTCACGAATTTGCGT	55.6			
